# Protective Effects of a Standardized Water Extract from the Stem of *Ipomoea batatas* L. Against High-Fat Diet-Induced Obesity

**DOI:** 10.3390/nu17101643

**Published:** 2025-05-12

**Authors:** Chae-Won Lee, Ye Seul Yoon, Young-Seo Yoon, Kyung-Sook Chung, Mi-ju Kim, Geonha Park, Minsik Choi, Young-Pyo Jang, Kyung-Tae Lee

**Affiliations:** 1 Department of Fundamental Pharmaceutical Science, Graduate School, Kyung Hee University, Seoul 02447, Republic of Korea; codnjs963@khu.ac.kr; 2 Department of Pharmaceutical Biochemistry, College of Pharmacy, Kyung Hee University, Seoul 02447, Republic of Korea; 3 Development Department, Dalim Biotech Co., Ltd., Wonju-si 26348, Gangwon-do, Republic of Korea; plan783@dalimpharm.co.kr (Y.S.Y.); miju0522@dalimpharm.co.kr (M.-j.K.); 4 BELABEL BIO. Inc., Gimhae-si 50969, Gyeongsangnam-do, Republic of Korea; shapira@naver.com (Y.-S.Y.); adella76@hanmail.net (K.-S.C.); 5 Division of Pharmacognosy, College of Pharmacy, Kyung Hee University, Seoul 02447, Republic of Korea; ginapark0326@khu.ac.kr (G.P.); alstlr7595@naver.com (M.C.); 6 Department of Oriental Pharmaceutical Sciences, College of Pharmacy, Kyung Hee University, Seoul 02447, Republic of Korea; 7 Department of Biomedical and Pharmaceutical Sciences, Graduate School, Kyung Hee University, Seoul 02447, Republic of Korea; 8 Department of Integrated Drug Development and Natural Products, Graduate School, Kyung Hee University, Seoul 02447, Republic of Korea

**Keywords:** *Ipomoea batatas* L., anti-obesity, AMP-activated protein kinase, white-adipose tissue, liver, lipogenesis, brown adipose tissue, thermogenesis, microbiota

## Abstract

**Background/Objectives:** Obesity is a major health concern that can lead to various chronic diseases. Little is known about the anti-obesity effect of a standardized hot water extract from the stems of *Ipomoea batatas* (WIB). This study aimed to evaluate the therapeutic potential of WIB as a natural alternative to conventional anti-obesity treatments by assessing its effects on body weight, fat accumulation, and key metabolic biomarkers in a high-fat diet-induced obesity model. **Methods:** A high-fat diet (HFD) induced obesity in C57BL/6 mice. The mice were then treated orally with either orlistat (positive control) or WIB. Changes in body weight, food intake, and fat weight were measured, along with blood lipid profiles and adipokines. Western blot analyses were conducted to determine protein levels in each tissue. H&E staining in white adipose tissue and liver, and the gut microbiota composition were analyzed. **Results:** WIB treatment significantly reduced body weight and fat mass compared to the HFD group and demonstrated comparable effects to orlistat. WIB improved blood lipid profiles and adipokine levels. H&E staining revealed reduced fat accumulation in the white adipose tissue and liver. Also in those tissues, WIB restored expression levels of sterol regulatory element-binding protein-1 (SREBP-1) and CCAAT/enhancer-binding protein α (C/EBPα) and increased AMP-activated protein kinase (AMPK) phosphorylation. In brown adipose tissue, WIB enhanced AMPK phosphorylation and upregulated thermogenic-related proteins, including peroxisome proliferator-activated receptor-gamma coactivator-1α (PGC-1α), peroxisome proliferator-activated receptor α (PPARα), sirtuin 1 (SIRT1), uncoupling protein-1 (UCP-1), and cytochrome C oxidase subunit 4 (COX-IV). Analysis of gut microbiota revealed that WIB normalized β-diversity and reversed HFD-induced phyla imbalances (notably in *Bacteroidetes*, *Firmicutes*, and *Proteobacteria*). **Conclusions:** By reducing adiposity under the conditions tested in a murine model, improving metabolic markers, and favorably modulating gut microbiota, WIB demonstrates potential in mitigating obesity-related risks. These findings suggest that WIB may serve as a promising natural substance for the management of obesity. Further studies are warranted to confirm its efficacy and explore the potential underlying mechanisms in overweight or obese humans as a health supplement to help manage or prevent obesity.

## 1. Introduction

Globally, obesity is a major issue associated with several chronic ailments, such as heart disease, diabetes, and non-alcoholic fatty liver disease [1,2]. Because obesity is classified as a medical condition, appropriate treatment strategies should be implemented to mitigate obesity-associated health risks.

The relationship between obesity and adipose tissue is significant because adipose tissue plays a crucial role in the progression and development of obesity [3]. When a person eats too much food and has leftover caloric intake, excess energy is stored as fat in adipose tissue. Adipose tissue can be categorized into two primary types. White adipose tissue (WAT) functions as an energy reservoir. On the other hand, brown adipose tissue (BAT) contributes to energy consumption by generating heat [4]. Numerous studies and reviews have highlighted that adipose tissue acts as an active metabolic organ involved in systemic energy regulation and inflammation. Its dysfunction is closely associated with obesity and metabolic disorders [5,6]. Several transcription factors control lipogenesis. CCAAT/enhancer-binding protein alpha (C/EBPα) and sterol regulatory element-binding protein-1 (SREBP-1) are major transcription factors that regulate adipose lipid metabolism, with C/EBPα controlling adipocyte development and insulin sensitivity, and SREBP-1 regulating fatty acid and triglyceride synthesis in response to insulin [7]. The AMP-activated protein kinase (AMPK) has a crucial role in cellular energy balance and regulating adipocyte metabolism by controlling transcription factors, such as C/EBPα and SREBP-1, making it a stunning target of anti-obesity therapeutic strategies [8]. In BAT, AMPK and sirtuin 1 (SIRT1) activate peroxisome proliferator-activated receptor-gamma coactivator-1α (PGC-1α) by phosphorylation and deacetylation, respectively [9]. Activation of PGC-1α coactivates the peroxisome proliferator-activated receptor α (PPARα) and induces the expression of thermogenic genes like uncoupling protein 1 (UCP-1) [10]. Additionally, PGC-1α enhances the expression of cytochrome c oxidase IV (COX-IV) [11]. Because these signaling pathways are important for the regulation of thermogenesis, energy expenditure, and fat oxidation, BAT helps to counteract obesity and metabolic disorders [12].

The gut is an important organ for absorbing nutrients from digested foods, and the gut microbiota can influence this absorption process. *Proteobacteria*, *Firmicutes*, and *Bacteroidetes* are obesity-related bacterial phyla that affect energy regulation and fat storage [13,14]. In particular, studies have shown that obese individuals tend to have a higher ratio of *Firmicutes* to *Bacteroidetes* [15,16]. For these reasons, it is important to consider the restoration of the balance of gut microbes in obesity. Many studies have shown that intestinal microbiota participate in host energy metabolism through a range of metabolites and molecules, such as short-chain fatty acids (SCFAs), to fight obesity with bacteria [17,18]. Among these SCFAs, butyrate has been reported to serve as the main source of energy for intestinal epithelial cells, and it can improve fatty acid oxidation and reduce fat accumulation by regulating the AMPK signaling pathway [19].

In general, synthetic weight-loss drugs exhibit limited long-term efficacy, necessitating careful monitoring of the risk of adverse effects that may outweigh the therapeutic benefits in some individuals [20]. Orlistat was selected as the positive control drug in our animal experiments to compare its efficacy in reducing body weight and fat accumulation in obese animal models because it is a well-established anti-obesity medication that acts as a pancreatic lipase inhibitor and effectively reduces dietary fat absorption [21,22]. This selection allowed us to assess whether WIB exerts anti-obesity effects through mechanisms distinct from orlistat, thereby providing valuable insights into its potential therapeutic role. However, orlistat can cause adverse effects on the digestive system, including oily stools, flatulence, and increased bowel movement frequency [23]. The limitations of many synthetic weight-loss drugs imply growing interest in natural compounds with anti-obesity properties [24,25].

Sweet potatoes (*Ipomoea batatas*, Convolvulaceae) have been traditionally cultivated in many countries. Typically, only the tuber—the part most consumed—is utilized, while the stem is used only to a limited extent in countries like Korea. Consequently, the utilization of other parts of the sweet potato plants, such as the leaves and stems, remains restricted. Although several studies have demonstrated that the leaves and polyphenols of *I. batatas* showed anti-oxidant, anti-hyperglycemia, and anti-inflammatory effects, little is known about the anti-obesity effect from the stem of *I. batatas* [26,27,28,29]. Therefore, as a part of our ongoing screening program to evaluate the anti-obesity potential of natural products, we aimed to clarify the competence and molecular mechanisms underlying the anti-obesity properties of a standardized water extract from the stem of *Ipomoea batatas* L. (WIB) in a high-fat diet-induced mouse model.

## 2. Materials and Methods

### 2.1. Materials

The water used for the extraction was prepared using an ABBOTA NEO water purification system (UMC Science Co., Ilsan, Gyeonggi-do, Republic of Korea). HPLC-grade water, acetonitrile, and methanol were supplied by Fisher Scientific Korea (Seoul, Republic of Korea), and formic acid was purchased from Wako Pure Chemical Industries (Osaka, Japan). The substances used for preparing standard solutions, including L-tryptophan, neochlorogenic acid, chlorogenic acid, cryptochlorogenic acid, hyperoside, isoquercetin, isochlorogenic acid A, isochlorogenic acid B, and isochlorogenic acid C, were all supplied by ChemFaces (Wuhan, China), and caffeic acid was supplied by Sigma-Aldrich (Steinheim, Germany). JEOL’s YOKUDELNA calibration kit (Tokyo, Japan) was used for the calibration of the ESI-TOF-MS chromatogram and spectra, and high-purity nitrogen gas from Shinyang Oxygen Co. (Seoul, Republic of Korea) was utilized for MS analysis. Orlistat (O4139) was purchased from Sigma-Aldrich (St. Louis, MO, USA). Diets (D12450B and D12492) were purchased from Research Diets Inc. (New Brunswick, NJ, USA). C/EBPα (8178), phospho-AMPKα (2531), AMPKα (2532), SIRT1 (9475), and COX-IV (4844) antibodies were purchased from Cell Signaling Technology (Danvers, MA, USA). PGC-1α (sc-518025), PPARα (sc-398394), UCP-1 (sc-293418), and β-actin (sc-81178) antibodies were obtained from Santa Cruz Biotechnology (Santa Cruz, CA, USA). The SREBP-1 (ab28481) antibody was purchased from Abcam (Cambridge, MA, USA).

### 2.2. WIB Preparation, Identification, and Standardization

*Ipomoea batatas* L. was cultivated in Yeoju (Republic of Korea) and was identified by Prof. Young Pyo Jang (Division of Pharmacognosy, College of Pharmacy, Kyung Hee University, Republic of Korea). A voucher specimen has been deposited in Darim Biotech (Seoul, Republic of Korea). The stems of *Ipomoea batatas* L. were washed, air-dried, and then oven-dried at 50 °C for 30 h to remove moisture. The dried stems were ground, and the fine powder obtained by passing through an 850 μm sieve was used. For extraction, 10 g of this fine powder was mixed with 300 mL of distilled water and refluxed at 100 °C for 2 h. The extract was then filtered, and the filtrate was freeze-dried to prepare the respective extracts. For UPLC-PDA analysis, the *Ipomoea batatas* L. stem extract powder was dissolved in HPLC-grade water at a concentration of 30 mg/mL and subjected to ultrasonic extraction for 10 min. The extract was then filtered through a 0.2 μm Polyvinylidene Fluoride (PVDF) syringe filter (Whatman International Ltd., Maidstone, Kent, UK) to obtain the hot water extract solution. Standard solutions were prepared by dissolving L-tryptophan, neochlorogenic acid, caffeic acid, chlorogenic acid, cryptochlorogenic acid, hyperoside, isoquercetin, isochlorogenic acid A, isochlorogenic acid B, and isochlorogenic acid C in methanol at a concentration of 1 mg/mL. For UPLC-PDA-ESI-TOF-MS analysis of the components in *Ipomoea batatas* L. stems, an Acquity™ H-class UPLC system with PDA and Empower 3 Software (version Service Release 1, Waters Corp., Milford, MA, USA) was used. Separation was achieved using a Kinetex^®^ 1.7 μm EVO C18 100 Å 100 × 2.1 mm column and an EVO C18 guard column (Phenomenex, Torrance, CA, USA) with a flow rate of 0.3 mL/min. The column and autosampler temperatures were set at 25 °C and 20 °C, respectively. The injection volume was 2 μL, and the PDA detection wavelength was 254 nm. The mobile phase consisted of acetonitrile (A) and water (B) with 0.1% formic acid, and the gradient was as follows: A was maintained at 0% for 3 min, increased to 5% at 8 min, to 10% at 15 min, and finally to 25% at 35 min. For stabilization, A was increased to 95% at 40 min, held for 2 min, then decreased to 0% at 45 min and held for 5 min. The mass analysis of the extracts and standard solutions was performed using a JEOL Ltd. (Tokyo, Japan) JMS-T100TD (AccuTOF-TLC) with an electrospray ionization source. The mass range was 50 to 1000 *m*/*z*, and the positive mode conditions were as follows: orifice 1 voltage, 40 V; ring lens voltage, 15 V; orifice 2 voltage, 7 V; orifice 1 temperature, 80 °C; desolvation temperature, 250 °C; nitrogen gas flow for nebulizer and desolvation, 1 L/min and 3 L/min; needle electrode voltage, 2000 V; and peak voltage, 1000 V. Other conditions, including those for the negative mode, are detailed in Table S1.

### 2.3. Animals and Experimental Design

C57BL/6J mice (male, 5–6 weeks old, 21 ± 1 g) were obtained from Orient Bio Inc. (Seongnam, Republic of Korea). Mice were kept under standard conditions (light–dark cycle, 12 h; temperature, 22 ± 1 °C; humidity, 50 ± 10%) for a week. All procedures adhered to the Kyung Hee University Animal Care and Use Guidelines, and the experimental protocol was approved by the University’s Experimentation Committee (KHSASP-22-215, Approval date: 22 July 2022). Mice were divided into 5 groups (*n* = 8) to examine the effect of WIB: vehicle-treated control (CON) group, HFD group for obesity, orlistat (20 mg/kg) group for positive control, and WIB (100 or 300 mg/kg) group. Each group was housed in a cage. The formula compositions of the diet are summarized in Table 1. Based on these diets, the CON group received a normal diet, while the rest of the group was fed a high-fat diet for 10 weeks to induce obesity. At the same time, each group received daily oral administration of vehicle, orlistat, or WIB using a zonde (8 cm, 15G, Duksan general science, Seoul, Republic of Korea). The weight of the mice and their food intake were measured once a week. At the end of the experiment, the mice were euthanized by 70% CO_2_, and tissues were collected immediately after surgical sacrifice under sterile conditions. After dissection, the tissues were carefully excised and rinsed with PBS to remove any residual blood. They were subsequently stored in sterilized tubes at −80 °C until further analysis to ensure sample integrity.

### 2.4. Body Fat Composition Analysis

Before sacrifice, the body fat composition of each group was measured using dual-energy X-ray absorptiometry (DEXA) (InAlyzer, Medikors, Seongnam, Republic of Korea). The lean tissue, fat tissue, and differentiated tissue from lean to fat were exposed to blue, red, and green/yellow, respectively. The amount of fat in the tissue was determined and represented as grams.

### 2.5. H&E Staining Analysis

After sacrifice, WAT and liver obtained from the mice were fixed with 4% formaldehyde solution overnight. The fixed tissues were made into paraffin blocks, and the slices of paraffin blocks were stained with hematoxylin and eosin (H&E). The stained tissue sections were examined under a light microscope system (Cell Sense standard ver.1.9, Olympus corporation, Tokyo, Japan).

### 2.6. Protein Extraction and Western Blot Analysis

Total protein was extracted from adipose tissues and the liver with the protein extraction solution PRO-PREP (Intron Biotechnology, Seoul, Republic of Korea). The debris was removed by centrifuge at 4 °C for 30 min, and the supernatants were carefully transferred into a new tube. The protein concentration was quantified with the Bradford assay. A total of 30 μg of whole proteins were resolved by SDS-PAGE on 8–15% polyacrylamide gel and transferred electrically onto a PVDF membrane. The immunoblots were then incubated overnight with a 1:1000 diluted primary antibody at 4 °C, washed three times with TBS/T, incubated with a 1:2000 diluted secondary antibody for 2 h at room temperature, and washed three times again with TBS/T. The blots were finally developed using an ECL chemiluminescence substrate (Santa Cruz Biotechnology, Santa Cruz, CA, USA). The protein bands were visualized using Amersham Hyperfilm ECL (GE Healthcare Life Sciences, Chicago, IL, USA).

### 2.7. Biochemical Examination of Blood Plasma

During the sacrifice, blood samples were collected from a vein and stored in tubes coated with heparin. After collecting blood samples, they were centrifuged at 2500 rpm at room temperature for 10 min. The separated plasmas were stored at −80 °C. Plasma levels of total cholesterol (TC), triglycerides (TG), low-density lipoprotein (LDL), high-density lipoprotein (HDL), very-low-density lipoprotein (VLDL), GOT, GPT, BUN, insulin, leptin, and adiponectin were measured to evaluate common lipid parameters, hepatotoxicity, nephrotoxicity, and obesity-related hormonal status. Each parameter was analyzed using an AU480 chemistry analyzer (Beckman Coulter, Brea, CA, USA) from T&P Bio (Gwangju, Republic of Korea).

### 2.8. Genomic DNA (gDNA) Extraction and Microbiome Profiling

Total genomic DNA was extracted from the stool samples using a QIAamp^®^ Fast DNA Stool Mini Kit (Qiagen, Hilden, Germany) according to the manufacturer’s instructions. The V3–V4 regions of the bacterial 16S ribosomal RNA (rRNA) gene were amplified using barcode-indexed 515F and 806R primers. The 16S rRNA gene amplicons were detected by electrophoresis on 1.5% agarose gels, purified with a ProNex^®^ Size-Selective Purification System (Promega, Madison, WI, USA), and quantified with a QuantiFluor^®^ One dsDNA System (Promega, Madison, WI, USA). All samples were pooled in equivalent concentration for producing a sequencing library and the library was quantified at 2 nM using KAPA Library Quantification Kit Illumina^®^ platforms (Roche, Basel, Switzerland). Finally, pyrosequencing was performed using an Illumina iSeq 100 platform (Illumina Inc., San Diego, CA, USA).

### 2.9. Statistical Analysis

Data are presented as mean ± standard error of the mean (SEM). One-way ANOVA with Duncan’s test was used to assess differences between groups (SPSS, Chicago, IL, USA). *p* < 0.05 was regarded as representing statistical significance, and for multiple comparisons, statistical differences among the groups are indicated below using ‘a, b, c, and d’.

## 3. Results

### 3.1. UPLC-PDA-ESI-TOF-MS for Identification of WIB Constituents

**Figure 1:**
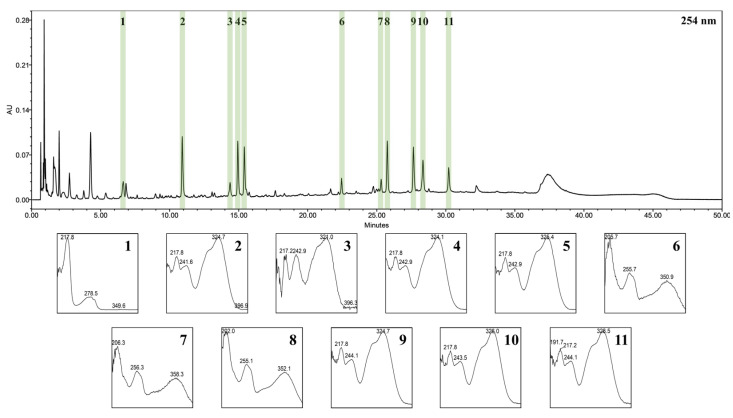
The UPLC-PDA chromatogram and peak-specific UV spectrum of identified WIB compounds; (**1**) L-tryptophan, (**2**) neochlorogenic acid, (**3**) caffeic acid, (**4**) chlorogenic acid, (**5**) cryptochlorogenic acid, (**6**) quercetin 3-O-*β*-D-sophoroside, (**7**) hyperoside, (**8**) isoquercetin, (**9**) isochlorogenic aicd B, (**10**) isochlorogenic acid A, (**11**) isochlorogenic acid C.

WIB (141.83 g) was obtained from 500 g of dried *I. batatas* stem powder, with a yield of 28.366%. UPLC-PDA-ESI-TOF-MS analysis successfully identified 11 key components of WIB. Figure 1 displays the characteristic chromatogram observed at 254 nm and UV spectrum of each peak. Table 2 outlines the essential information for each compound, including retention time (Rt) at 254 nm, precursor ions, mass-to-charge ratio (*m*/*z*), elemental composition, and mass differences (mmu). All compounds were confirmed through direct comparison with reference standard compounds, except for peak 6. This particular peak was tentatively identified as quercetin-3-*O*-*β*-D-sophoroside based on a review of the existing literature [30].

### 3.2. WIB Lessens the Body Weight Gain in HFD-Induced Mice

**Figure 2:**
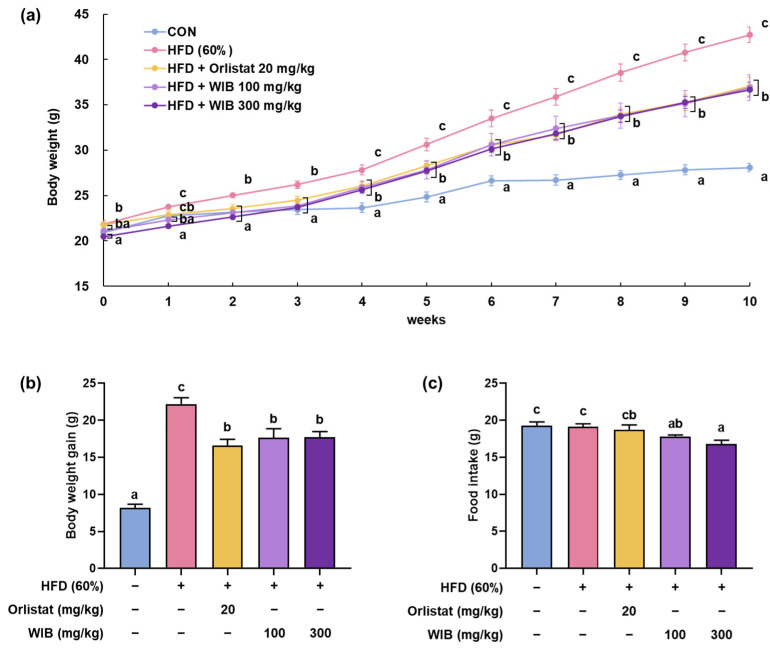
Repressive effects of WIB on body weight in HFD-induced obese mice. HFD-induced mice were administered the indicated doses of WIB (100 and 300 mg/kg) daily during the experiment. (**a**) Body weights, (**b**) total body weight gain for 10 weeks, and (**c**) food intake in 1 week (*n* = 8). Values are represented as the mean ± standard error of the mean (SEM). Values with different letters are significantly different, *p* < 0.05 (a < b < c).

Five experimental groups were established to investigate the effect of WIB on the body weight of HFD-fed mice. Over a 10-week period, the HFD group exhibited a significant increase in body weight compared with the CON group. From the third week onward, the orlistat-administered group (positive control) and the WIB group exhibited lower body weights than the HFD group (Figure 2a). Especially, body weight on the final day of the experiment (HFD group: 42.72 ± 0.85 g vs. WIB 100 mg/kg group: 36.90 ± 1.20 g; WIB 300 mg/kg group: 36.67 ± 0.78 g) and body weight gain (HFD group: 22.21 ± 2.24 g vs. WIB 100 mg/kg group: 17.67 ± 3.17 g; WIB 300 mg/kg group: 17.71 ± 2.06 g) were elevated in HFD group and lessened by WIB treatment. Orlistat treatment significantly decreased body weight and weight gain, and a similar pattern was observed in the WIB (100 and 300 mg/kg) treatment groups (Figure 2b). Food intake was measured to determine whether there was a change in appetite. WIB treatment significantly decreased the amount of food intake compared to the vehicle-treated control and HFD group (Figure 2c).

### 3.3. WIB Modified the Fat Composition in HFD-Induced Mice

**Figure 3:**
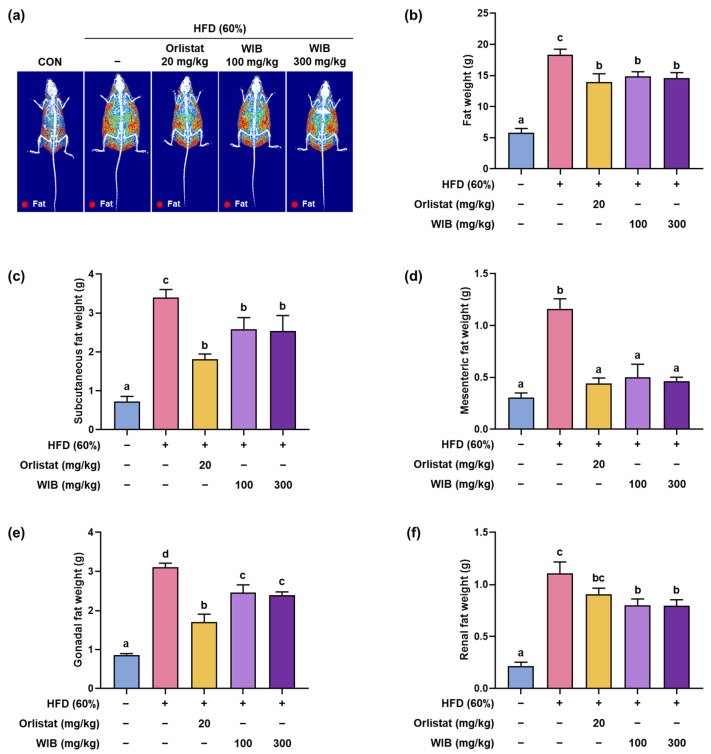
Effects of WIB on body composition and fat mass in HFD-induced obese mice. (**a**) Radiography images of fat tissues. (**b**) The fat weight in each group was measured through DEXA analysis. Fat was obtained from each tissue after sacrificing the mice, and their weights were measured. (**c**–**f**) Subcutaneous, mesenteric, gonadal, and renal fat weights. Values are represented as the mean ± SEM (DEXA analysis; *n* = 3, fat in tissue weight; *n* = 5). Values with different letters are significantly different, *p* < 0.05 (a < b < c < d).

To comprehensively verify the inhibitory effects of WIB on fat accumulation without invasive assessments, we employed DEXA analysis. HFD caused mice to accumulate more fat than in the CON group, and WIB treatment reduced fat weight in the whole body (HFD group: 18.38 ± 0.86 g vs. WIB 100 mg/kg group: 14.86 ± 0.75 g; WIB 300 mg/kg group: 14.57 ± 0.90 g, *p* < 0.05) (Figure 3a,b). Among the various adipose tissue deposits, subcutaneous fat is the most susceptible to dietary modulation. Accordingly, we analyzed the accumulation of fat deposits in subcutaneous tissue, along with the mesenteric, gonadal, and renal tissue weights. Fat deposits gained by the HFD were significantly reduced by WIB treatment (Figure 3c–f). These results indicate that WIB-reduced fat accumulation in various body regions contributes to weight loss.

### 3.4. WIB Ameliorates the Lipid Parameters in Blood Plasma of HFD-Induced Mice

Obesity is associated with abnormal blood lipid levels. TC, TG, LDL, and VLDL levels were significantly higher in the HFD group than in the CON group (Table 3). The WIB 300 mg/kg and orlistat groups showed significantly reduced TG, LDL, and VLDL levels compared to the HFD group. Notably, WIB 100 mg/kg significantly decreased only LDL levels, while TG and VLDL levels showed a mild reduction without statistical significance. While only the orlistat group showed a statistically significant decrease in TC levels, the consistent, albeit non-significant, reduction observed in both WIB-treated groups suggests a potential lipid-lowering effect of WIB. Additionally, although HFD did not notably reduce HDL levels, orlistat treatment significantly increased HDL compared to the HFD group. A slight, non-significant increase in HDL levels was also observed in both WIB-treated groups.

### 3.5. WIB Refined the Obesity-Related Hormones in Blood Plasma of HFD-Induced Mice

**Figure 4:**
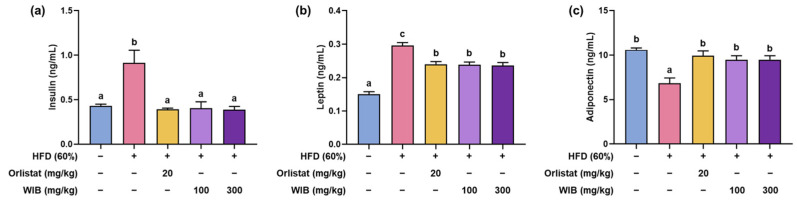
Effects of WIB on obesity-related hormones in HFD-induced obese mice. (**a**–**c**) Insulin, leptin, and adiponectin levels. Values are represented as the mean ± SEM (*n* = 5). Values with different letters are significantly different, *p* < 0.05 (a < b < c).

Adipokines, such as leptin, adiponectin, and insulin, can influence lipid profile, lipolysis, and lipogenesis [31]. Compared with the CON group, the secretion of insulin and leptin in the HFD group was surpassed, and adiponectin was curtailed (Figure 4a–c). However, the altered leptin, adiponectin, and insulin levels were significantly normalized by WIB treatment.

### 3.6. WIB Reduced Hypertrophy of Lipid Droplets and Lipogenesis in Subcutaneous Tissue of HFD-Induced Mice

**Figure 5:**
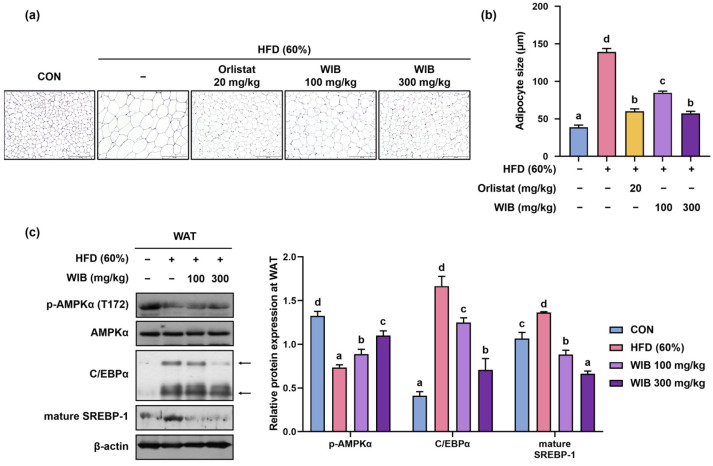
Effects of WIB on adipocyte size and lipogenesis in subcutaneous fat tissues of HFD-induced obese mice. (**a**) Histological analysis of subcutaneous fat tissue via H&E staining at ×20 magnification. (**b**) Representative diameters of adipocytes in the tissue. (**c**) Levels of p-AMPKα and lipogenic transcription factors were determined by Western blot analysis. Arrowed bands are the main band of that protein. Quantitative values for the Western blot of p-AMPKα to AMPKα, CEBP/α, and SREBP-1 are shown. β-Actin was used as an internal control. Values are represented as the mean ± SEM (adipocyte size; *n* = 5, density; *n* = 3). Values with different letters are significantly different, *p* < 0.05 (a < b < c < d).

Lipid parameters and adipocytes are closely linked because they play a central role in lipid storage [32]. To verify the effect of WIB on lipid accumulation in WAT, subcutaneous fat pads were visualized using H&E staining. The size of adipocytes was expanded in HFD mice and decreased significantly in orlistat and WIB-treated mice (HFD group: 139.18 ± 4.55 μm, WIB 100 mg/kg group: 84.74 ± 2.23 μm, *p* < 0.05, WIB 300 mg/kg group: 57.31 ± 2.82 μm, *p* < 0.05) (Figure 5a,b). Based on the histological analysis, phosphorylation of AMPKα and expression of C/EBPα and SREBP-1 were analyzed by Western blot (Figure 5c). Phosphorylation of AMPKα (Thr 172) was potently suppressed in the HFD group and significantly recovered by WIB treatment. However, WIB treatment did not alter the total AMPKα expression levels, indicating that only AMPKα phosphorylation was affected. In addition, WIB treatment significantly reduced the expression of C/EBPα and mature SREBP-1, which was notably increased in the HFD group.

### 3.7. WIB Prevents Lipid Accumulation in Liver Tissue of HFD-Induced Mice

**Figure 6:**
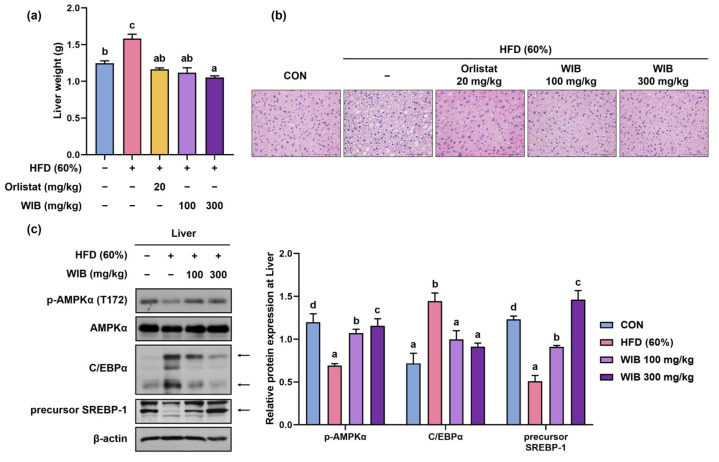
Effects of WIB on lipid accumulation in the liver tissues of HFD-induced obese mice. (**a**) Comparison of liver weights of each group. (**b**) The images of H&E-stained liver tissues at ×40 magnification. (**c**) Expression levels of p-AMPKα and lipogenic transcription factors were determined by Western blot analysis. Arrowed bands are the main band of that protein. Quantitative values for the Western blot of p-AMPKα to AMPKα, CEBP/α, and SREBP-1 are shown. β-Actin was used as an internal control. Values are represented as the mean ± SEM (liver weight; *n* = 5, density; *n* = 3). Values with different letters are significantly different, *p* < 0.05 (a < b < c < d).

Liver weight can indirectly indicate lipid accumulation in the hepatic tissues [33]. The weight of the liver increased in the HFD group and significantly decreased in orlistat or WIB treatment group (HFD group: 1.58 ± 0.06 g, WIB 100 mg/kg group: 1.12 ± 0.07 g, *p* < 0.05, WIB 300 mg/kg group: 1.05 ± 0.02 g, *p* < 0.05) (Figure 6a). To confirm whether the increase in liver weight was due to lipid accumulation, liver fat was visualized by H&E staining. The liver tissue of the HFD group showed fat accumulation, whereas the administration of orlistat or WIB prevented this amassment (Figure 6b). Based on the histological analysis and the liver weight, phosphorylation of AMPKα and expression of C/EBPα and SREBP-1 were analyzed by Western blot. In accordance with the findings observed in subcutaneous fat, WIB treatment recovered the HFD-induced downregulation of phosphorylated AMPKα (Thr 172) in the liver. The HFD group exhibited increased levels of C/EBPα, which were subsequently normalized by the WIB treatment. The precursor SREBP-1 showed reduced expression in the HFD group; however, its expression was restored upon WIB treatment (Figure 6c).

### 3.8. WIB Upregulates the Levels of Thermogenic-Related Proteins in BAT of HFD-Induced Mice

**Figure 7:**
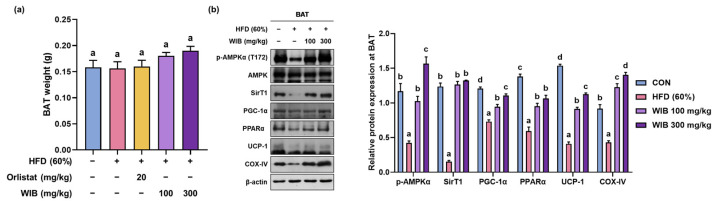
Effect of WIB on thermogenesis-related factors in the BAT of HFD-induced obese mice. (**a**) Weight of BAT in each group. (**b**) Expression levels of p-AMPKα, AMPKα, and thermogenesis-related proteins were determined by Western blot analysis. Quantitative values for Western blot are shown in graphs. β-Actin was used as an internal control. Values are represented as the mean ± SEM (BAT weight; *n* = 5, density; *n* = 3). Values with different letters are significantly different, *p* < 0.05 (a < b < c < d).

BAT is involved in thermogenesis by increasing energy expenditure via the utilization of stored fat [34]. Although no group attained statistically significant changes in BAT weight compared to the CON group, WIB treatment mildly increased BAT weight (Figure 7a). After the WIB treatment, the ratio of p-AMPKα/AMPKα was significantly elevated compared to the HFD group. The expression levels of key thermogenesis-related factors such as PGC-1α, PPARα, SIRT1, UCP-1, and COX-IV were significantly restored compared to those in the HFD group (Figure 7b).

### 3.9. WIB Ameliorates the Dysbiosis by Regulating the Gut Microbiota Composition in HFD-Induced Mice

**Figure 8:**
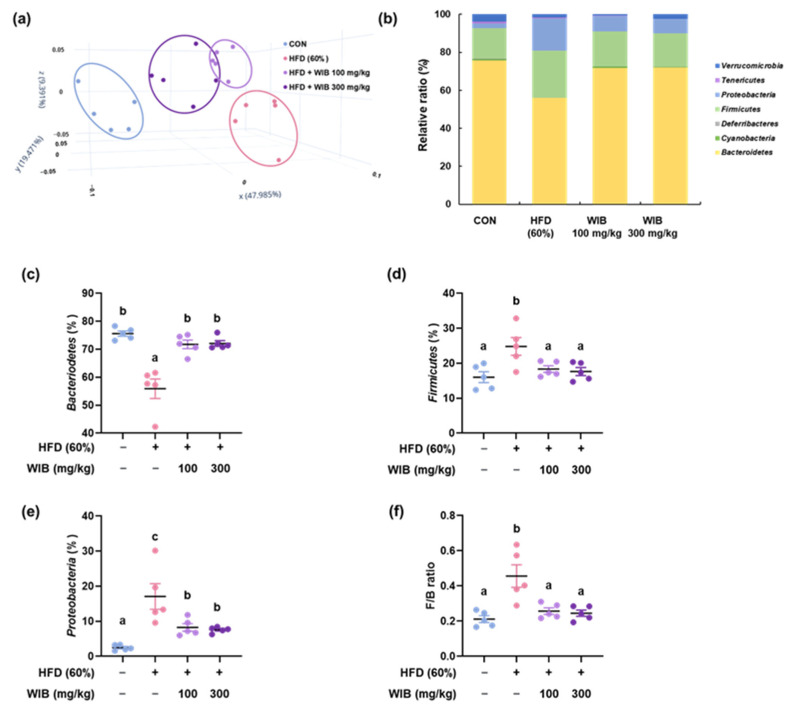
Regulatory effects of WIB on microbiota composition and colonization. (**a**) β-Diversity between groups was analyzed using a PCoA plot. (**b**) Phylum-level composition ratios in each group. (**c**–**f**) Relative ratios of *Bacteroidetes*, *Firmicutes*, and *Proteobacteria* and F/B ratio. Values are represented as the mean ± SEM (*n* = 5). Values with different letters are significantly different, *p* < 0.05 (a < b < c).

Obesity is often associated with unbalanced or disrupted gut microbiota, characterized by reduced microbial diversity and altered ratios of key bacterial groups [35]. This dysbiosis is associated with increased fat storage and metabolic disturbances [36]. Principal coordinate analysis (PCoA) plots based on β-diversity were used to examine the differences in the phylogenetic relationships of microbial communities. The plot indicated that the microbial community of the HFD group shifted from that of the CON group, whereas the WIB-treated groups were located at a distance from the HFD group, which was closer to that of the CON group (Figure 8a). Based on these results, the microbiome composition at the phylum level was analyzed for each group. Figure 8b shows that compared to the CON group, the phylum-level composition shifted in the HFD group, but was restored to resemble that of the CON group with WIB treatment. In the HFD group, *Bacteroidetes* levels decreased compared to those in the CON group but increased with WIB treatment (Figure 8c). Conversely, *Firmicutes* and *Proteobacteria* levels were upregulated in the HFD group compared to those in the CON group but downregulated with WIB treatment (Figure 8d,e). The F/B ratio was significantly higher in the HFD group than that in the CON group. However, WIB treatment ameliorated this increase relative to that observed in the HFD group (Figure 8f).

## 4. Discussion

Obesity is an excess body fat accumulation, which can cause several health problems related to hypertension, hyperlipidemia, atherosclerosis, and type 2 diabetes and can lead to heart attacks and strokes [37]. The well-known causes of obesity are genetic factors, high-calorie food intake, reduced physical activity, and psychological factors [38]. Currently, anti-obesity medications such as semaglutide offer satisfactory weight loss, but injections once a week may be less acceptable to patients. Additionally, its side effects include nausea, vomiting, and diarrhea, which may prevent patients from being consistent with the medication [39]. This indicates the demand for new agents that can alter metabolic processes with fewer side effects than pharmaceuticals [40]. Therefore, this study aimed to develop new functional food candidates for the prevention of obesity by leveraging the weight-reducing potential of standardized natural products. In this regard, we focused on the potential use of sweet potato stems, an edible natural resource, to develop functional materials aimed at reducing body fat.

Sweet potatoes are starchy root crops that are cultivated worldwide because of their nutritive value and taste [41]. Research on the stems of sweet potatoes has been less extensive than that on tubers. According to some studies, sweet potato leaves are known to contain high amounts of polyphenolic compounds such as flavonols, catechins, hydroxycinnamic acids, and hydroxybenzoic acids, as well as triterpenes, alkaloids, anthraquinones, and coumarins [42,43]. Each compound is known to have anti-oxidant, antidiabetic, and/or anti-inflammatory effects [44,45]. In particular, we identified quercetin 3-*O*-*β*-D-sophoroside as one of the constituents of WIB from sweet potato stems. Quercetin is well known for its anti-obesity effects; therefore, we hypothesized that WIB may possess anti-obesity properties [46]. To test this, we investigated the weight-reducing effects and molecular mechanisms of WIB in C57BL/6 mice fed HFD.

Fat accumulation accompanying obesity refers to the excessive storage of fat in the adipose tissue of the body [47]. First, we monitored overall weight changes to verify the inhibitory effects of WIB on fat accumulation. Despite the expectation that weight loss would be greater in the 300 mg/kg WIB group than in the 100 mg/kg WIB group, this decrease was similar in both groups. This is because, within the body, drugs can act non-linearly or become saturated above a certain concentration, leading to inconsistent increases in efficacy with increasing concentrations [42]. DEXA was used to measure the total fat distribution to quantify fat reduction. We analyzed fat weight reduction in various tissues to observe local effects. The findings illustrate how WIB contributes to the reduction in both total body fat and fat in specific tissues.

Blood analysis was conducted to analyze toxicity levels, lipid profiles, and hormone concentrations. Toxicity analysis revealed that WIB administration did not affect liver and kidney function, meaning that the observed weight loss in WIB was not caused by toxicity (Figure S1). Upregulated blood levels of TG, VLDL, and LDL occur when the liver stores excess fatty acids that cannot be efficiently processed or utilized [48,49]. As WIB restored normal level of TG, VLDL, and LDL, it is plausible that WIB ameliorates hepatic steatosis without adversely affecting liver function. There are many obesity-related hormones; however, only the key hormones, insulin, leptin, and adiponectin, were measured. Insulin regulates TG synthesis and lipogenesis in adipose tissue [50]. However, persistent HFD promotes insulin resistance, resulting in increased insulin secretion, but insulin becomes unable to perform its normal function [51]. Adiponectin can improve insulin sensitivity, and leptin reduces body weight and fat by inhibiting appetite [52]. Analysis of hormone levels revealed that HFD caused abnormal changes in insulin, leptin, and adiponectin levels, but these changes were recovered by WIB treatment. In particular, the reduced leptin secretion was reflected in a corresponding decrease in food intake. By restoring the hormone and blood lipid biomarkers linked to obesity, WIB is thought to decrease the development of metabolic syndrome and its associated chronic conditions.

Based on the widely accepted view that WAT secretes a diverse array of peptide and steroid hormones, including leptin, cytokines, and adiponectin, it is evident that obesity-induced adipokine dysregulation is closely linked to subcutaneous fat [53]. Alterations in the secretion profile of these adipokines from subcutaneous adipose tissue may significantly contribute to the development of metabolic disturbances. AMPK, a key cellular energy sensor, plays a regulatory role in protein, lipid, and glucose metabolism, and its activation downregulates SREBP-1 expression, thereby inhibiting lipogenesis [54,55]. SREBP-1 can regulate PPARγ, which in turn activates C/EBPα [56]. C/EBPα activates metabolic adipocyte genes, and SREBP-1 regulates lipogenesis [57]. WIB can inhibit fat accumulation by modulating the transcriptional level of lipogenesis through AMPK activation, exhibiting its potential against obesity.

Our bodies attempt to cope with excess caloric intake by burning fat [58]. BAT is essential for thermogenic activity and contributes to energy expenditure [59]. In BAT, AMPK also acts as a regulator of energy balance. BAT employs mitochondria for ATP synthesis and heat production, with UCP-1 playing a key role by uncoupling respiration from ATP production to generate heat, thus combating obesity [60]. It is well known that COX-IV supports oxidative phosphorylation. AMPKα and SIRT-1 enhance PGC-1α activity. PGC-1α is involved in the regulation of thermogenic genes, activation of PPARα, and induction of UCP-1 expression, which in turn promotes lipolysis [12,61]. Our data showed that the WIB administration increased BAT weight and regulated the expression of thermogenesis-related proteins, including SIRT1, UCP-1, PGC-1α, PPARα, and COX-IV in BAT of HFD-induced obese mice.

Although AMPK signaling is primarily regulated within individual tissues, it is plausible that enhanced AMPK activity in subcutaneous fat could influence liver metabolism through the secretion of adipokines and metabolites that act as inter-tissue communicators. Moreover, AMPK promotes the beigeing process of WAT by regulating energy metabolism, highlighting its pivotal role in linking WAT and BAT [62]. Taken together, these findings suggest that AMPK acts as a central metabolic hub connecting the liver, WAT, and BAT, thereby coordinating systemic energy homeostasis. This coordinated AMPK activation may underlie the comprehensive metabolic improvements observed with WIB treatment, providing a mechanistic basis for its anti-obesity effects.

The gut microbiota can affect obesity management [36]. Elevated levels of *Proteobacteria* can influence host energy balance by modifying the absorption and storage of dietary energy [63]. *Firmicutes* exhibit high efficiency in energy metabolism, influencing the absorption of calories [64]. Obese individuals typically have a greater abundance of *Proteobacteria* and *Firmicutes* in their gut microbiomes than non-obese individuals. In contrast, *Bacteroidetes* are known to be easily found in nonobese individuals. The differentially abundant Firmicutes and Bacteroidetes also play a critical role in obesity [7]. Significant changes in the F/B ratio denote gut microbiota dysbiosis. F/B ratios are linked to energy uptake and storage, and they provide critical insights into strategies that can be employed to prevent obesity via modulation of the microbiome [65]. As the treatment of WIB normalized the microbiome composition and F/B ratio, WIB has the potential to modulate gut microbiome composition and prevent dysbiosis. Based on our findings, we propose that the bioactivity of WIB is mediated through AMPK activation, which modulates adipose tissue formation and liver metabolism, ultimately contributing to the inhibition of lipogenesis and anti-obesity effects [66,67]. For further investigation, we examined microbiota, which is generally known to be associated with obesity [13,68,69] and found that WIB treatment significantly restored HFD-induced dysbiosis. Recently, Zhang et al. identified that intestinal AMPKα1 stimulates thermogenesis by modulating antimicrobial peptides that, in turn, influence gut microbiota and metabolites [70]. Based on this correlation, it can be inferred that WIB exerts a comprehensive effect on obesity.

Although in vivo experiments have provided valuable insights into the anti-obesity effects of WIB, the underlying molecular mechanisms have not been directly validated through in vitro assays. In addition, definitive target validation at the protein or receptor level was not conducted, which limits mechanistic interpretation. Further studies using cell-based models and target-specific approaches are warranted to confirm the signaling pathways involved. Moreover, we recognize the necessity for further investigation to assess the applicability of WIB to human subjects.

## 5. Conclusions

This study visualized the anti-obesity effects of WIB in HFD-induced mice. Reductions in body weight and fat mass, with advanced blood lipid profiles and adipokines, were achieved by WIB. Furthermore, WIB induced the down-regulation of p-AMPKα expression, fat accumulation, and the expression of key lipogenic transcription factors in the liver and WAT. It affects BAT by enhancing energy expenditure and metabolism-regulated protein expression. WIB’s ability to modulate the gut microbiota resulted in the effect of WIB on obesity-related gut dysbiosis. These findings support the potential use of WIB as a natural therapeutic agent for obesity management.

## Figures and Tables

**Table 1:** Formula composition of a normal and high-fat diet.

Comparative Profiles	Normal Diet		High-Fat Diet	
	g%	kcal%	g%	kcal%
Protein	19.2	20	26	20
Carbohydrate	67.3	70	26	20
Fat	4.3	10	35	60
Total		100		100
kcal/g	3.85		5.24	

**Table 2:** Retention time (Rt), precursor ion, elemental composition, and mass difference in the identified peaks of WIB.

Peak No.	Rt (min)	Precursor Ion *(m*/*z)*	Elemental Composition (or Molecular Formula)	Mass Difference (mmu)	Identification
1	6.6	205.09655 [M+H]^+^ 203.07701 [M−H]^−^	C_11_H_12_N_2_O_2_	−1.16 −5.04	L-tryptophan ^1^
2	10.8	355.10230 [M+H]^+^ 377.08625 [M+Na]^+^ 353.08623 [M−H]^−^	C_16_H_18_O_9_	−0.61 1.39 −1.03	Neochlorogenic acid ^1^
3	14.	181.04995 [M+H]^+^ 179.03368 [M−H]^−^	C_9_H_8_O_4_	−0.13 −0.75	Caffeic acid ^1^
4	14.9	355.10254 [M+H]^+^ 377.08614 [M+Na]^+^ 353.08607 [M−H]^−^	C_16_H_18_O_9_	−0.36 1.29 −1.18	Chlorogenic acid ^1^
5	15. 3	355.10254 [M+H]^+^ 377.08647 [M+Na]^+^ 353.08644 [M−H]^−^	C_16_H_18_O_9_	−0.36 1.62 −0.82	Cryptochlorogenic acid ^1^
6	22.3	627.16048 [M+H]^+^ 625.14879 [M−H]^−^	C_27_H_30_O_17_	4.35 8.31	Quercetin 3-*O*-*β*-D-sophoroside
7	25.1	465.10362 [M+H]^+^ 463.08995 [M−H]^−^	C_21_H_20_O_12_	0.32 2.30	Hyperoside ^1^
8	25.6	465.10496 [M+H]^+^ 463.08931 [M−H]^−^	C_21_H_20_O_12_	1.66 1.66	Isoquercetin ^1^
9	27.4	517.13593 [M+H]^+^ 539.11944 [M+Na]^+^ 515.12501 [M−H]^−^	C_25_H_24_O_12_	1.33 2.89 6.06	Isochlorogenic acid B ^1^
10	28.1	517.13653 [M+H]^+^ 539.11977 [M+Na]^+^ 515.12431 [M−H]^−^	C_25_H_24_O_12_	1.93 3.23 5.36	Isochlorogenic acid A ^1^
11	30.0	517.13486 [M+H]^+^ 515.12623 [M−H]^−^	C_25_H_24_O_12_	0.26 7.28	Isochlorogenic acid C ^1^

^1^ Identified using reference standard compounds.

**Table 3:** Effects of WIB on lipid parameters in HFD-induced obesity mice.

Variable (mg/dL)	CON	HFD	Orlistat (20 mg/kg)	WIB (100 mg/kg)	WIB (300 mg/kg)
TC	103.43 ± 14.88 ^a^	158.71 ± 8.04 ^c^	125.71 ± 37.84 ^ab^	148.57 ± 23.14 ^bc^	147.29 ± 10.73 ^bc^
TG	52.86 ± 9.91 ^a^	115.14 ± 15.60 ^c^	66.57 ± 25.25 ^ab^	86.43 ± 31.72 ^bc^	68.00 ± 42.49 ^ab^
LDL	9.71 ± 2.14 ^a^	15.29 ± 1.25 ^c^	11.57 ± 2.64 ^ab^	12.86 ± 2.04 ^b^	11.57 ± 2.23 ^ab^
HDL	86.57 ± 5.91 ^a^	91.14 ± 14.24 ^a^	97.71 ± 13.83 ^b^	96.00 ± 9.07 ^ab^	99.57 ± 8.79 ^ab^
VLDL	8.86 ± 1.65 ^a^	18.29 ± 2.70 ^c^	10.12 ± 2.29 ^ab^	14.40 ± 5.29 ^bc^	11.21 ± 6.93 ^ab^

Values are represented as the mean ± SEM (*n* = 8). Values with different letters are significantly different, *p* < 0.05 (a < b < c).

## Data Availability

The original contributions presented in this study are included in the article/Supplementary Materials; further inquiries can be directed to the corresponding author.

## Supplementary Materials

The following supporting information can be downloaded at: https://www.mdpi.com/article/10.3390/nu17101643/s1, Table S1: Positive and negative ion mode ESI-TOF-MS conditions for identification of WIB components; Figure S1: The blood plasma levels of GOT, GPT, and BUN. Values with the same letter are not significantly different.
