# Supplementary figures and images for: Protective Effects of a Standardized Water Extract from the Stem of Ipomoea batatas L. Against High-Fat Diet-Induced Obesity
Source: Nutrients. 2025 May 12;17(10):1643. doi: 10.3390/nu17101643 (PMC12113841; doi:10.3390/nu17101643)

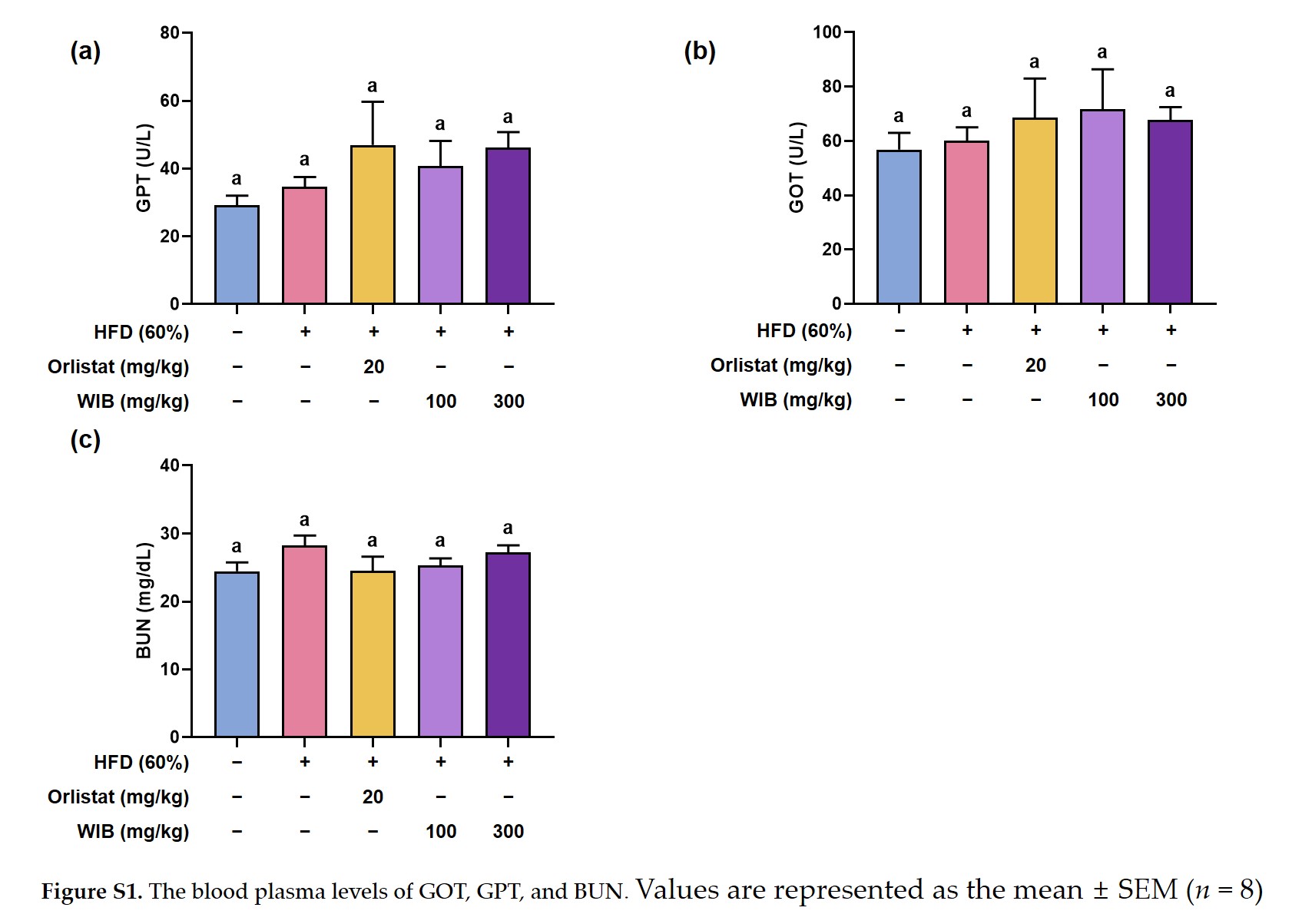

Supplement: Supplementary file 1 [file nutrients-17-01643-s001.zip › Figure S1.jpg]

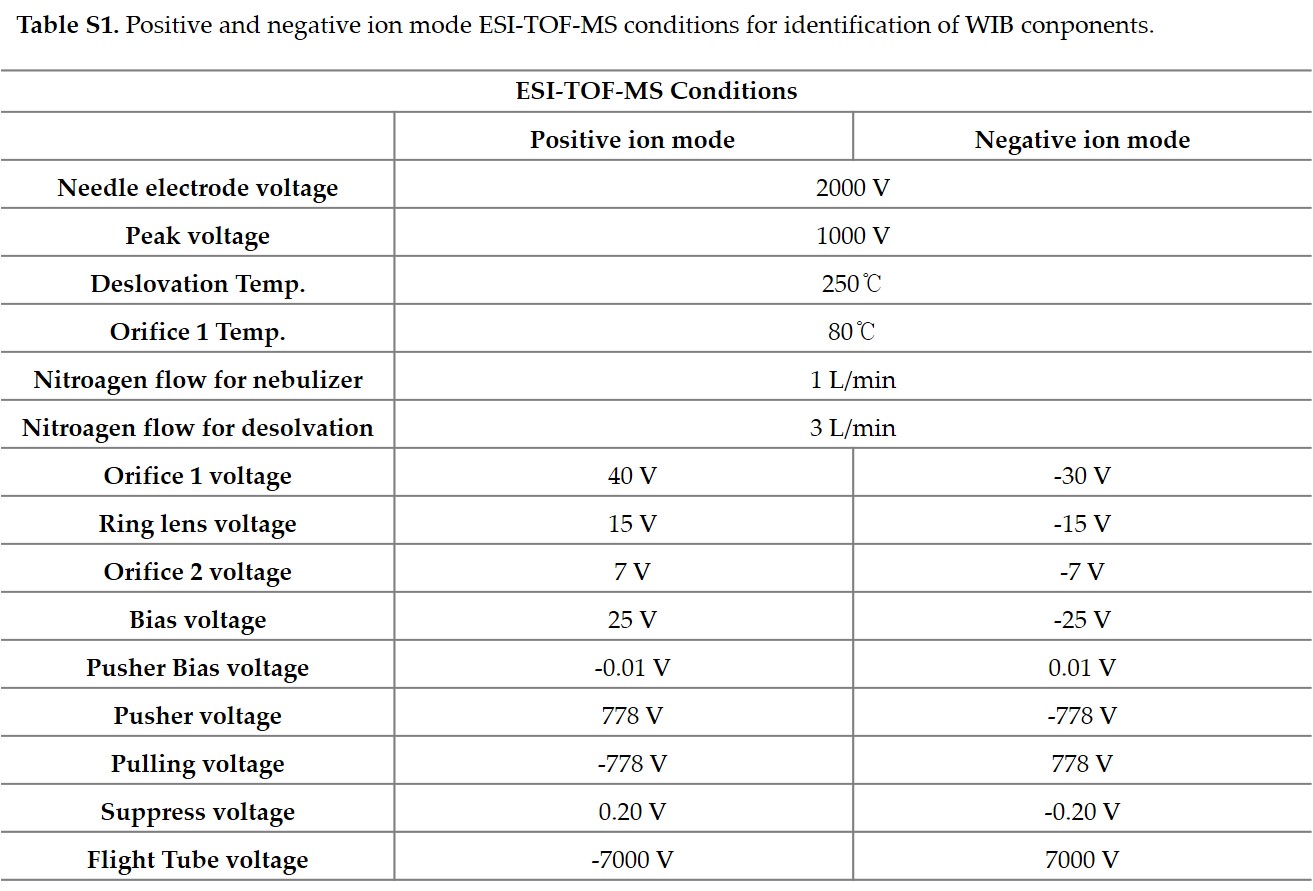

Supplement: Supplementary file 1 [file nutrients-17-01643-s001.zip › Table S1.jpg]
